# Alterations of Dermal Connective Tissue Collagen in Diabetes: Molecular Basis of Aged-Appearing Skin

**DOI:** 10.1371/journal.pone.0153806

**Published:** 2016-04-22

**Authors:** Angela J. Argyropoulos, Patrick Robichaud, Rebecca Mutesi Balimunkwe, Gary J. Fisher, Craig Hammerberg, Yan Yan, Taihao Quan

**Affiliations:** 1 Department of Psychiatry, University of Washington, Seattle, Washington, United States of America; 2 Department of Dermatology, University of Michigan Medical School, Ann Arbor, Michigan, United States of America; 3 Department of Dermatology, Plastic Surgery Hospital, Chinese Academy of Medical Sciences & Peking Union Medical College, Beijing, China; San Gallicano Dermatologic Institute, ITALY

## Abstract

Alterations of the collagen, the major structural protein in skin, contribute significantly to human skin connective tissue aging. As aged-appearing skin is more common in diabetes, here we investigated the molecular basis of aged-appearing skin in diabetes. Among all known human matrix metalloproteinases (MMPs), diabetic skin shows elevated levels of MMP-1 and MMP-2. Laser capture microdissection (LCM) coupled real-time PCR indicated that elevated MMPs in diabetic skin were primarily expressed in the dermis. Furthermore, diabetic skin shows increased lysyl oxidase (LOX) expression and higher cross-linked collagens. Atomic force microscopy (AFM) further indicated that collagen fibrils were fragmented/disorganized, and key mechanical properties of traction force and tensile strength were increased in diabetic skin, compared to intact/well-organized collagen fibrils in non-diabetic skin. In *in vitro* tissue culture system, multiple MMPs including MMP-1 and MM-2 were induced by high glucose (25 mM) exposure to isolated primary human skin dermal fibroblasts, the major cells responsible for collagen homeostasis in skin. The elevation of MMPs and LOX over the years is thought to result in the accumulation of fragmented and cross-linked collagen, and thus impairs dermal collagen structural integrity and mechanical properties in diabetes. Our data partially explain why old-looking skin is more common in diabetic patients.

## Introduction

Diabetes affects every organ system of the body including the skin [1]. It is estimated that more than two-thirds (79.2%) of diabetic patients experience a skin problem at some stage throughout the course of their disease [2]. Many of these skin conditions can occur in anyone, but are acquired more easily in diabetics [1–3]. For example, in comparison to the general population, diabetic patients more commonly experience aged-appearing skin [1, 2, 4] as well as more skin infections such as those secondary to foot ulcers [5, 6]. In fact, such skin problems are sometimes the first warning indicator for internal complications of diabetes and may allow an astute physician to initiate diagnostic testing.

In non-diabetic human skin, old-looking skin is known to be caused by fragmentation of dermal connective tissue, collagen [7, 8], the major structural protein responsible for skin's firmness [9]. Fragmentation of collagen fibrils is a prominent feature of aged human skin [10], which severely impairs skin structural integrity and mechanical properties. It is well-documented that age-associated elevation of matrix metalloproteinase (MMP) is largely responsible for fragmentation of collagen fibrils in non-diabetic aged human skin [7, 8, 10].

Although molecular alterations in non-diabetic aging skin are relatively well-characterized, not much is known about the alteration of MMPs and dermal collagen structural and mechanical properties in diabetic human skin. Here, we investigated the expression of all known human MMPs [11], LOX, and nanoscale morphology and mechanical properties of collagen fibrils in diabetic human skin. We found that diabetic skin shows elevated levels of MMP-1 and MMP-2, and LOX, which may contribute to increased fragmentation and cross-linking of the collagen fibrils. Fragmented and cross-linked collagen impairs dermal collagen structural integrity resulting in alterations of mechanical properties in diabetic skin. These data demonstrate the molecular basis of aged-appearing skin in diabetes, which partially explain why old-looking skin is more common in diabetic patients.

## Materials and Methods

### Human skin samples

Research involving human subjects was approved by the University of Michigan Institutional Review Board, and all subjects provided written informed consent. Diabetic skin (45–62 years) and age-matched normal human skin samples were obtained by punch biopsy (4 mm) from sun-protected underarm, as described previously [12]. Based on previous data that we have collected from human skin biopsies in similar studies [8, 10, 12], we collected a sample size of N = 12 in order to detect a two-fold change with an expected probability of at least 90%, using a two-tailed test for group comparisons at the 0.05 level of significance, assuming an expected variance of 1.12. Study exclusion criteria include that all human subjects were HIV negative, and none of the subjects had any systemic or autoimmune diseases, nor were they being treated with steroids or hormonal therapy. All diabetic patients included in the study carried the diagnosis of type 2 diabetes, and all had no significant skin disorders, such as foot ulcers, psoriasis, and dermatitis, and no the history of smoking. Individuals with diabetes mellitus type 1 were excluded.

### Laser capture microdissection

For laser capture microdissection (LCM), human skin samples embedded in optimal cutting temperature (OCT) compound, and were sectioned (15 μm). The skin sections were stained with hematoxylin and eosin. Epidermis and dermis were captured by LCM (Leica ASLMD system; Leica Microsystems, Wetzlar, Germany), as described previously [13]. Total cellular RNA was extracted from LCM-captured epidermis and dermis using an RNeasy® Micro Kit (Qiagen, Chatsworth, CA, USA) according to the manufacturer’s instructions. The quality and quantity of total RNA were determined by Agilent 2100 bioanalyzer (Agilent Technologies, Santa Clara, CA, USA).

### Cell culture and zymography

Adult primary human skin fibroblasts were cultured from normal human skin biopsies by the procedure described previously [14]. Briefly, full-thickness punch biopsies (4mm) were obtained from adult buttock skin. The dermis was separated from epidermis by trypsinization (0.25% trypsin,0.1% EDTA) for 30 minutes at 37°C in phosphate buffered saline. The biopsy was minced into small pieces and placed to tissue culture dish. Dulbecco’s modified minimal essential medium supplemented with nonessential amino acids and 10% fetal bovine serum (DMEM-FBS) was used as culture medium. Only a minimal amount of medium was added so that tissue pieces would adhere to the plastic surface. The dishes were maintained at 37°C in an atmosphere of 95% air and 5% CO_2_. The tissue was removed after one week, at which time cells were migrated out from the edge of the dermal tissue fragments. The cells were harvested with 0.25% trypsin/EDTA and grown in DMEM (Thermal Fisher Scientific, Waltham, MA), supplemented with 10% (vol/vol) fetal calf serum, 5 U/ml heparin, 100 IU/ml penicillin, and 0.1 mg/ml streptomycin. Medium was refreshed every 3 days. Cells were used between passages 3 and 9. To explore the effect of glucose concentration on the MMPs mRNA expression, cells were incubated in either low (5mM, 11885, Thermal Fisher Scientific, Waltham, MA) or high (25mM, 11665, Thermal Fisher Scientific, Waltham, MA) glucose DMEM media for 48 hours. For zymography assay, conditioned media from the cultured cells were concentrated and then analyzed by electrophoresis in the presence of 12% Zymogram (casein) protein gel (Thermal Fisher Scientific, Waltham, MA). After electrophoresis, the gel was incubated in Zymogram Renaturing buffer (Thermal Fisher Scientific, Waltham, MA) for 30min at room temperature with gentle agitation. After renaturing, the gel was incubated in Developing buffer (Thermal Fisher Scientific, Waltham, MA) at 37°C for overnight. The MMPs activities were visualized by staining Coommasie Blue R-250 (Thermal Fisher Scientific, Waltham, MA) solution. MMPs inhibitor (GM60001, Santa Cruz Biotechnology, CA) was used as specificity of MMPs-mediated proteolytic activity.

### RNA isolation and quantitative real-time RT-PCR

Total RNA from human skin and human skin dermal fibroblasts was prepared using TRizol reagent (Invitrogen, Carlsbad, CA) according to the manufacturer’s protocol. Total RNA from LCM captured tissues was extracted using RNeasy micro kit (Qiagen, Gaithersburg, MD, USA). cDNA for PCR templates was prepared by reverse transcription of total RNA (100 ng) using Taqman Reverse Transcription kit (Applied Biosystems, Carlsbad, CA, USA). Real-time PCR was performed on a 7700 Sequence Detector (Applied Biosystems, Carlsbad, CA, USA) using Taqman Universal PCR Master Mix Reagents (Applied Biosystems, Carlsbad, CA, USA). All real-time PCR primers were purchased from RealTimePrimers.com (Real Time Primers, LLC, Elkins Park, PA, USA). Target gene mRNA expression levels were normalized to the housekeeping gene 36B4 as an internal control for quantification.

### Western analysis

Whole cell proteins were prepared from human skin tissues and cells using WCE buffer (25mMHepes, 0.3MNaCl, 1.5 mMMgCl2, 0.2mMEDTA,1%Triton,20mMbeta-glycerol-phosphate), and protein levels were determined by Western analysis. Briefly, proteins from human skin dermis were resolved on 10% SDS-PAGE, transferred to PVDF membrane, and blocked with PBST (0.1% Tween 20 in PBS) containing 5% milk. Primary antibodies (MMP-1/MMP-2/ MMP-14, Chemicon International, Temecula, CA, USA; Col-1, RDI Research Diagnostics, Flanders, NJ, USA; Col-3, Santa Cruz Biotechnology, CA; LOX, Thermo Fisher Scientific, Rockford, IL, USA) were diluted in the PBST solution (1:200) and were incubated with PVDF membrane for one hour at room temperature. Blots were washed three times with PBST solution and incubated with appropriate secondary antibody for one hour at room temperature. After washing three times with PBST, the blots were developed with ECF (Vistra ECF Western Blotting System, Amersham Pharmacia Biotech, Piscataway, NJ, USA) following the manufacturer’s protocol. The membranes were scanned by STORM PhosphorImager (Molecular Dynamics, Sunnyvale, CA, USA), and the intensities of each band were quantified and normalized using β-actin as loading control.

### Atomic force microscopy (AFM) imaging

Nanoscale morphology and mechanical properties of the skin dermis were measured by AFM using previously established techniques in our laboratory with minor modifications [15]. Briefly, OCT embedded human skin samples were sectioned (50 μm) and mounted on glass coverslips (1.2 mm diameter, Fisher Scientific Co., Pittsburgh, PA). These AFM samples were allowed to air dry for at least 24 hours before AFM analysis. Mechanical properties; traction forces, tensile strength, and deformation were determined by Dimension Icon AFM system (Bruker-AXS, Santa Barbara, CA, USA) using PeakForce^TM^ Quantitative NanoMechanics mode using a silicon AFM probe (PPP-BSI, force constant 0.01–0.5N/m, resonant frequency 12-45kHz, NANOSENSORS™, Switzerland). PeakForce^TM^ Quantitative Nanomechanical Mapping (QNM^TM^) is a new AFM Nano-mechanical and Nano-imaging mode for measuring the Young's modulus of materials with high spatial resolution and surface sensitivity, by probing at the nanoscale. It maps and distinguishes between nanomechanical properties, including modulus and adhesion, while simultaneously imaging sample topography at high resolution. AFM was conducted at the Electron Microbeam Analysis Laboratory (EMAL), University of Michigan College of Engineering, and analyzed using Nanoscope Analysis software (Nanoscope Analysis v120R1sr3, Bruker-AXS, Santa Barbara, CA, USA).

### Measurement of total MMP activity assay

Biopsies were homogenized using frosted glass-on-glass Duall vessels in assay buffer containing a cocktail of protease inhibitors (pepstatin (10 mM), leupeptin (10 mM), soyabean trypsin inhibitor (1 mg/ml), trasylol (100 units/ml), Ca^2+^ (0·2 mM) and Mg^2+^(1 mM) (all from Sigma). Total MMP activity was measured as the ability to degrade a fluorescent peptide substrate (Mca-Pro-Leu-Gly-Leu-Dpa-Ala-Arg-NH2), which is cleaved by activated MMPs at the Gly-Leu site [16, 17]. The cleavage of the Gly-Leu bond releases the highly fluorescent Mca group from the internal quenching group Dpa. The samples and the peptide (25 mg/ml) were incubated for 3 h at 37°C and the MMP activity was determined on a fluorimeter (Perkin-Elmer LS50B, lex 328 nm and lem 393 nm). Incubation with a specific MMP inhibitor GM 6001 (1 mM) (Calbiochem) was performed in parallel for all samples as a control for non-specific degradation of the peptide. The reaction was stopped by addition of HCL (0·2 M). Enzyme activity was evaluated by assessment of changes in the fluorescence signals.

### Measurement of pepsin-resistant collagens by HPLC

Collagens for acid-soluble and pepsin-soluble/-insoluble fractions were prepared by established protocol [18]. Briefly, collagen extract was suspended in buffer (pH 7.5) and Pepsin (Promega, Madison, WI) was added to the solution at ratio of 1:10 (w/w), and mixed briefly, incubated overnight at 37°C. The reaction was stopped by heating at 95°C for 10 minutes, and analyzed for hydroxyproline content by HPLC, as previously described [19].

### Statistical analysis

Data are expressed as mean ± SEM. Comparisons were made with the paired *t*-test (two groups) or the repeated measures of ANOVA (more than two groups). Multiple pair-wise comparisons were made with the Tukey Studentized Range test. All p values are two-tailed, and considered significant when <0.05.

## Results

### Elevated expression of MMP-1 and MMP-2 in diabetic human skin dermis *in vivo*

**Fig 1:**
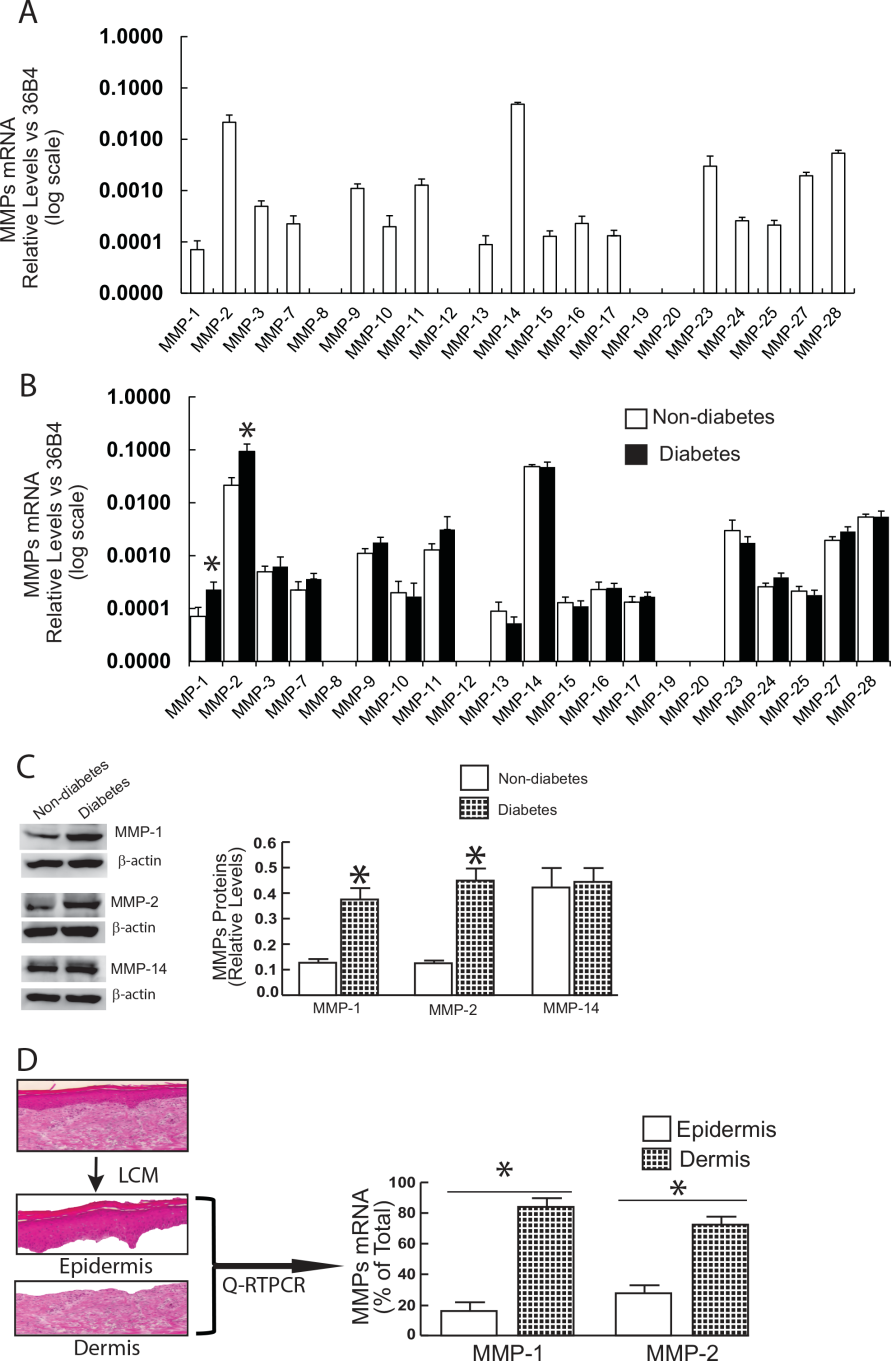
Elevated expression of MMP-1 and MMP-2 in diabetic human skin dermis *in vivo*. (A) MMP-1 and MMP-2 mRNA levels were elevated in diabetic skin relative to non-diabetic control skin. Total RNA was extracted from skin tissue and mRNA levels were quantified by real-time RT-PCR. MMPs mRNA levels were normalized to the housekeeping gene 36B4, as an internal control for quantification. Data are relative levels to 36B4 (mean±SEM). MMPs mRNA levels were expressed by log scale. N = 12, *p<0.05. (B) MMP-1 and MMP-2 protein levels were elevated in diabetic human skin. Protein levels were determined by Western analysis and normalized by β-actin (loading control). Insets (left panel) show representative Western blots. Data are expressed as mean±SEM, N = 6, *p<0.05. (C) Elevated MMPs in diabetic dermis. Epidermis and dermis were captured by Laser Capture Microdissection (LCM, see Methods for details). N = 6, *p<0.05. Total RNA was extracted from captured epidermis and dermis, and mRNA levels were quantified by real-time RT-PCR. MMPs mRNA levels were normalized to the housekeeping gene 36B4, as an internal control for quantification. Data are relative levels to 36B4 (mean±SEM). (D) MMP-1 and MMP-2, but not other MPs, were elevated in diabetic human skin dermis. Total RNA was extracted from the dermis captured by LCM and mRNA levels were quantified by real-time RT-PCR. MMPs mRNA levels were normalized to the housekeeping gene 36B4, as an internal control for quantification. Data are relative levels to 36B4 (mean±SEM). N = 6, *p<0.05.

We first compared the mRNA expression levels of all known MMPs in normal human skin and diabetic skin (Fig 1A). Among all known human MMPs, transcripts for MMP-8, -12, -19, and -20 were not detected. MMP-14 and -2 were the most highly expressed, while remaining MMPs were expressed at lower levels. Among the 19 MMPs expressed in human skin, MMP-1 (3.2-fold) and MMP-2 (4.4-fold) were significantly elevated in diabetic skin, compared to non-diabetic control skin (Fig 1A). Western blot further confirmed that MMP-1 and MMP-2 protein levels were elevated in diabetic skin but that MMP-14 levels were not elevated (Fig 1B). The relative levels of MMP-1 and MMP-2 in epidermis and dermis were determined by laser capture microdissection (LCM). Fig 1C shows that MMPs that were elevated in diabetic skin were primarily expressed in the dermis (MMP-1, 84%; MMP-2, 72%). LCM-coupled real-time PCR further confirmed that no other MMPs were elevated in the dermis of diabetic skin except MMP-1 and MMP-2 (Fig 1D). These data demonstrate MMP-1 and MMP-2 expression are significantly elevated in the dermis of diabetic skin.

### MMPs activity, tissue inhibitor of metalloproteinases (TIMPs), and collagen expression in diabetic human skin *in vivo*

**Fig 2:**
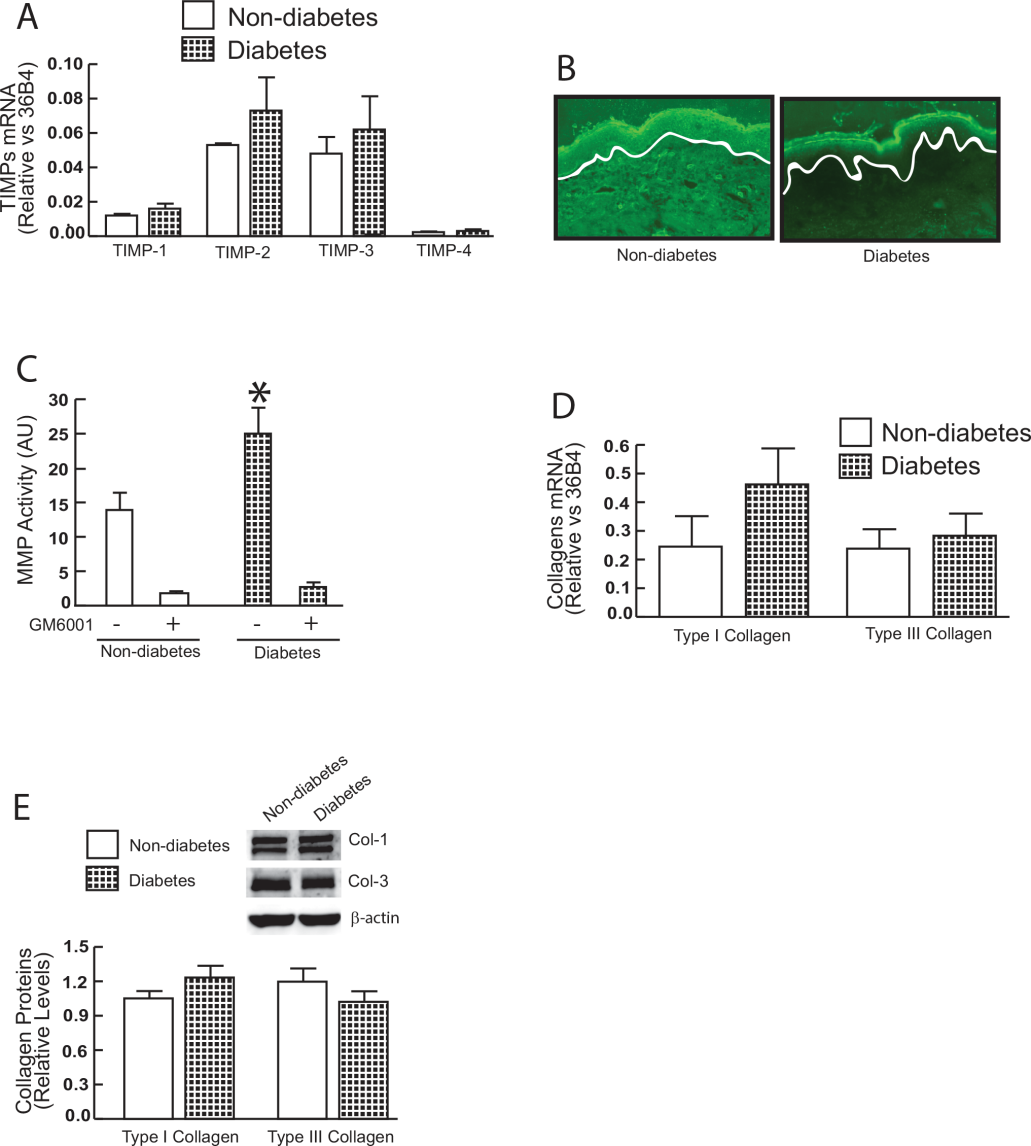
MMP activity, TIMPs, and collagen expression in diabetic human skin *in vivo*. (A) TIMP mRNA expression in diabetic and non-diabetic control skin. TIMP mRNA levels were normalized to the housekeeping gene 36B4, as an internal control for quantification. Data are relative levels to 36B4 (mean±SEM) N = 6. (B) Elevated MMP activity in the dermis of photodamaged forearm skin determined by in situ zymography. Loss of green fluorescence in diabetic dermis indicates degradation of fluorescein-collagen substrate. White lines indicate boundary between the epidermis (top) and dermis (bottom). N = 6. (C) MMP activity in diabetic and non-diabetic control skin (see Methods for details). N = 9–11. (D) Type I and type III procollagen mRNA expression in diabetic and non-diabetic control skin. Type I and type III procollagen mRNA levels were normalized to the housekeeping gene 36B4, as an internal control for quantification. Data are relative levels to 36B4 (mean±SEM) N = 6. (E) Type I and type III collagen protein levels were determined by Western analysis and normalized by β-actin (loading control). Insets (left panel) show representative Western blots. Data are expressed as mean±SEM, N = 6.

MMPs and TIMPs are often concurrently regulated as a means to control excess MMP activity. Therefore, we also investigated whether TIMPs are elevated in diabetic skin. We found that all four known TIMP genes (TIMP-1, -2, -3, and -4) are primarily expressed in human skin, however, no differences in mRNA levels of TIMP-1, -2, -3, or -4 were found between diabetic skin and non-diabetic control skin (Fig 2A). The observed preferential induction of MMPs (Fig 1) relative to TIMPs suggests that MMP activities are elevated in diabetic skin. To access MMP activity, we performed *in situ* zymography, in which unfixed skin sections are placed over a layer of fluorescently-labeled collagen. As shown in Fig 2B, elevated MMP activity in diabetic skin resulted in breakdown of the collagen, resulting in loss of fluorescence. Furthermore, the average activity of MMPs, measured as the ability to degrade a fluorescent peptide substrate (see Methods for details), was increased by 180% in diabetic skin compared to non-diabetic skin (Fig 2C). Increased MMP-1 expression in aged dermal fibroblasts is often accompanied with reduced collagen synthesis [8, 20]. Therefore, we evaluated the expression of type I and type III collagen, the major structural proteins in skin. These results revealed that mRNA (Fig 2D) and protein (Fig 2E) levels of type I and type III collagen were not altered in diabetics, compared to non-diabetic control skin. Taken together, these data demonstrate that MMP activity is significantly elevated, while there is no change in TIMP and collagen expression in diabetic skin.

### Collagen fibrils nanoscale morphology and mechanical properties in diabetic skin

**Fig 3:**
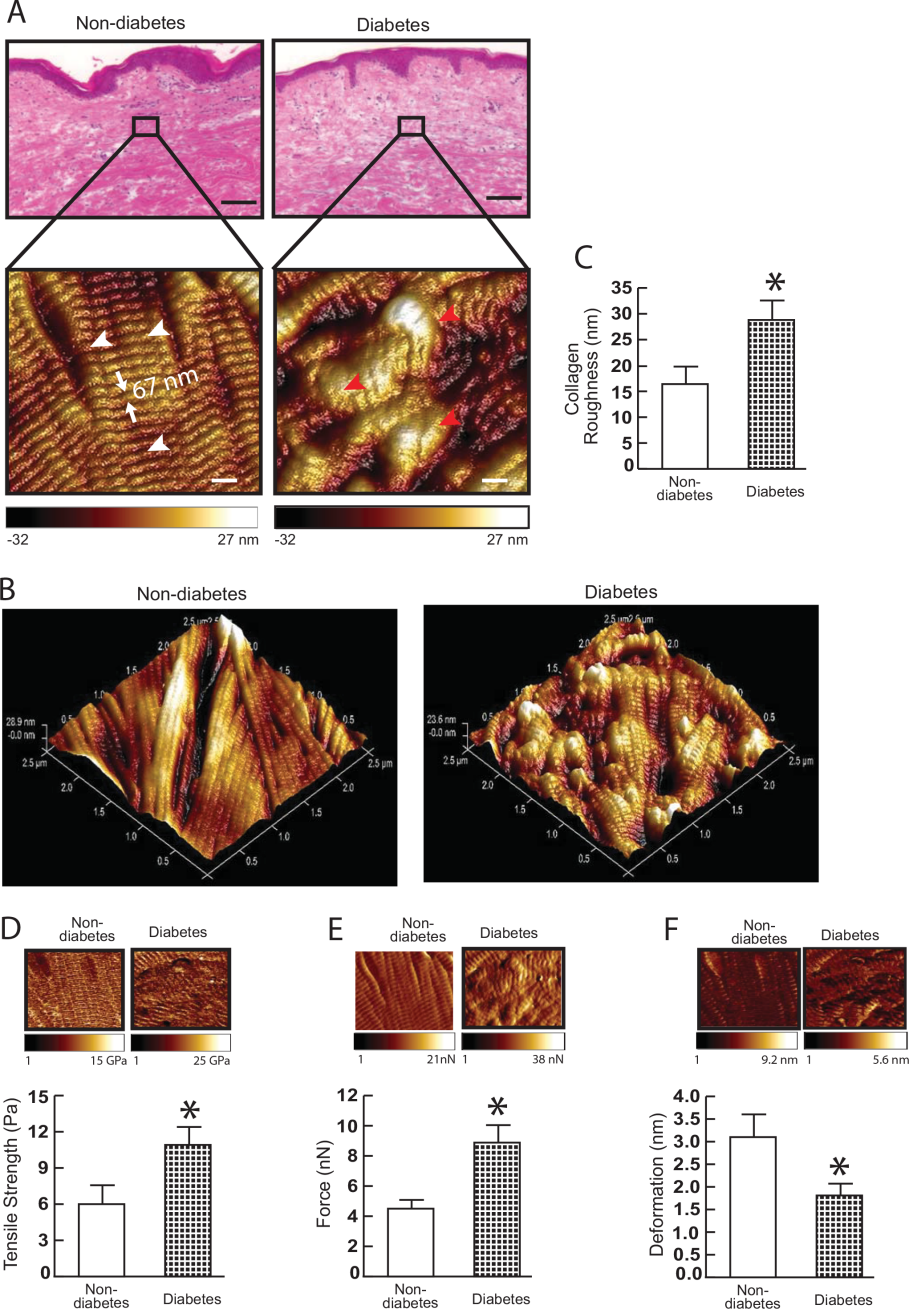
Collagen fibril nanoscale morphology and mechanical properties in diabetic skin. (A) Nanoscale collagen fibrils were imaged by atomic force microscopy (AFM). The white and red arrow heads indicate intact and fragmented/disorganized collagen fibrils, respectively. Images are representative of nine subjects. (B) Three-dimensional nanoscale collagen fibrils were imaged by AFM. Images are representative of nine subjects. (C) Collagen fibril roughness was analyzed using Nanoscope Analysis software (Nanoscope_Analysis_v120R1sr3, Bruker-AXS, Santa Barbara, CA). Results are expressed as the mean ± SEM, N = 6, *p<0.05. (D) Tensile strength, (E) traction forces, and (F) deformation were determined by AFM PeakForce^TM^ Quantitative NanoMechanics mode and Nanoscope Analysis software. Means±SEM. N = 8, *p<0.05.

As tissues’ mechanical properties regulate diverse cellular functions [15, 21, 22], we next employed atomic force microscopy (AFM) to compare mechanical properties of dermal collagen in diabetic and non-diabetic skin. We first determined the nanoscale morphology of collagen fibrils. AFM images revealed that collagen fibrils in non-diabetic dermis were intact, tightly packed and well organized (Fig 3A, left). In contrast, collagen fibrils in diabetic dermis were fragmented, sparse and disorganized (Fig 3A, right). Three-dimensional topographical AFM images further indicated that non-diabetic dermis was smooth and flattened (Fig 3B, left). In contrast, diabetic dermis was much rougher and more uneven (Fig 3B, right). Quantitative analysis of AFM data indicated that the average roughness (a measure of fibril organization) of dermal collagen fibrils in diabetic dermis was increased by 176% (Fig 3C, 29 nm vs. 16 nm), suggesting elevated MMPs may lead to fragmented and rough collagen fibrils. Fragmentation of collagen fibrils impairs dermal collagen structural integrity, and thus alters mechanical properties of the dermis. The key mechanical properties; traction force (Fig 3D) and tensile strength (Fig 3E), were increased by 182% and 197%, respectively, while collagen fibrils deformation was reduced by 58% (Fig 3F), in diabetic dermis compared to non-diabetic dermis. Taken together, these data demonstrate fragmentation and alterations of mechanical properties of dermal collagen fibrils in diabetic skin.

### Alteration of lysyl oxidase (LOX) expression diabetic skin

**Fig 4:**
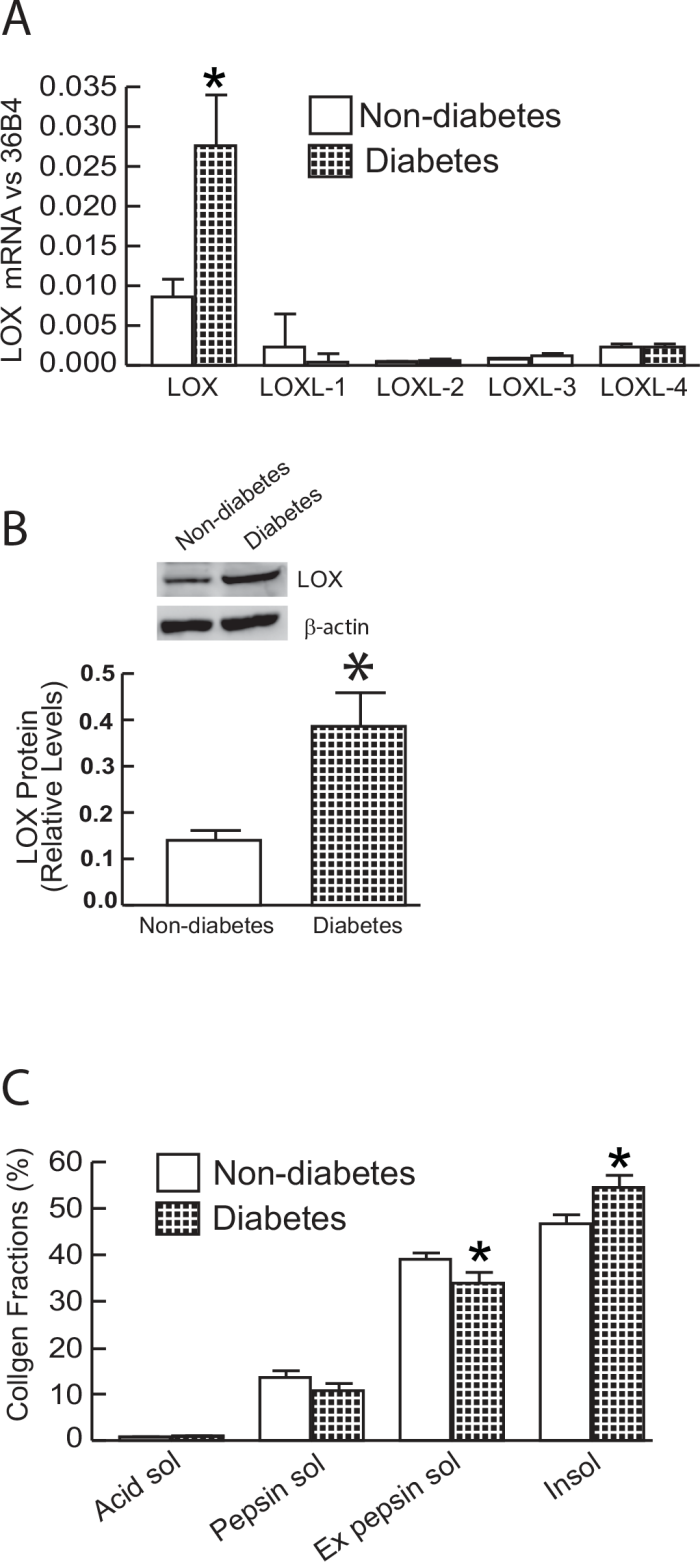
Alteration of lysyl oxidase (LOX) expression diabetic skin. (A) LOX family members mRNA expression in diabetic and non-diabetic control skin. Lox family mRNA levels were normalized to the housekeeping gene 36B4, as an internal control for quantification. Data are relative levels to 36B4. Mean±SEM. N = 6. (B) LOX protein levels were determined by Western analysis and normalized by β-actin (loading control). Insets (left panel) show representative Western blots. Data are expressed as mean±SEM, N = 6, *p<0.05. (C) Collagen solubility by pepsin digestion was determined by HPLC (see Methods for details). Mean±SEM. N = 12, *p<0.05.

Mechanical properties of collagen fibrils are also affected by collagen crosslinking, which is catalyzed by a lysyl oxidase (LOX)-mediated enzymatic process [23, 24]. Therefore, we determined five members of lysyl oxidases [LOX, LOX-like 1~4 (LOXL1~4)] mRNA expression in diabetic skin. Interestingly, among five LOX family members, LOX, which was most highly expressed in skin, was elevated in diabetic skin compared to non-diabetic skin (Fig 4A). Western blot further confirmed that LOX protein level was elevated in diabetic skin (Fig 4B). As cross-linked collagen resists proteolytic breakdown, we determined the susceptibility of collagen to pepsin digestion using HPLC. We found that pepsin-resistant insoluble collagens were increased, while pepsin soluble collagens were significantly reduced in diabetic skin in comparison to non-diabetic skin (Fig 4B). These data demonstrate that elevated LOX expression and higher cross-linked collagens exist in diabetic skin.

### High glucose increases MMPs expression and proteolytic activity in primary human skin dermal fibroblasts

**Fig 5:**
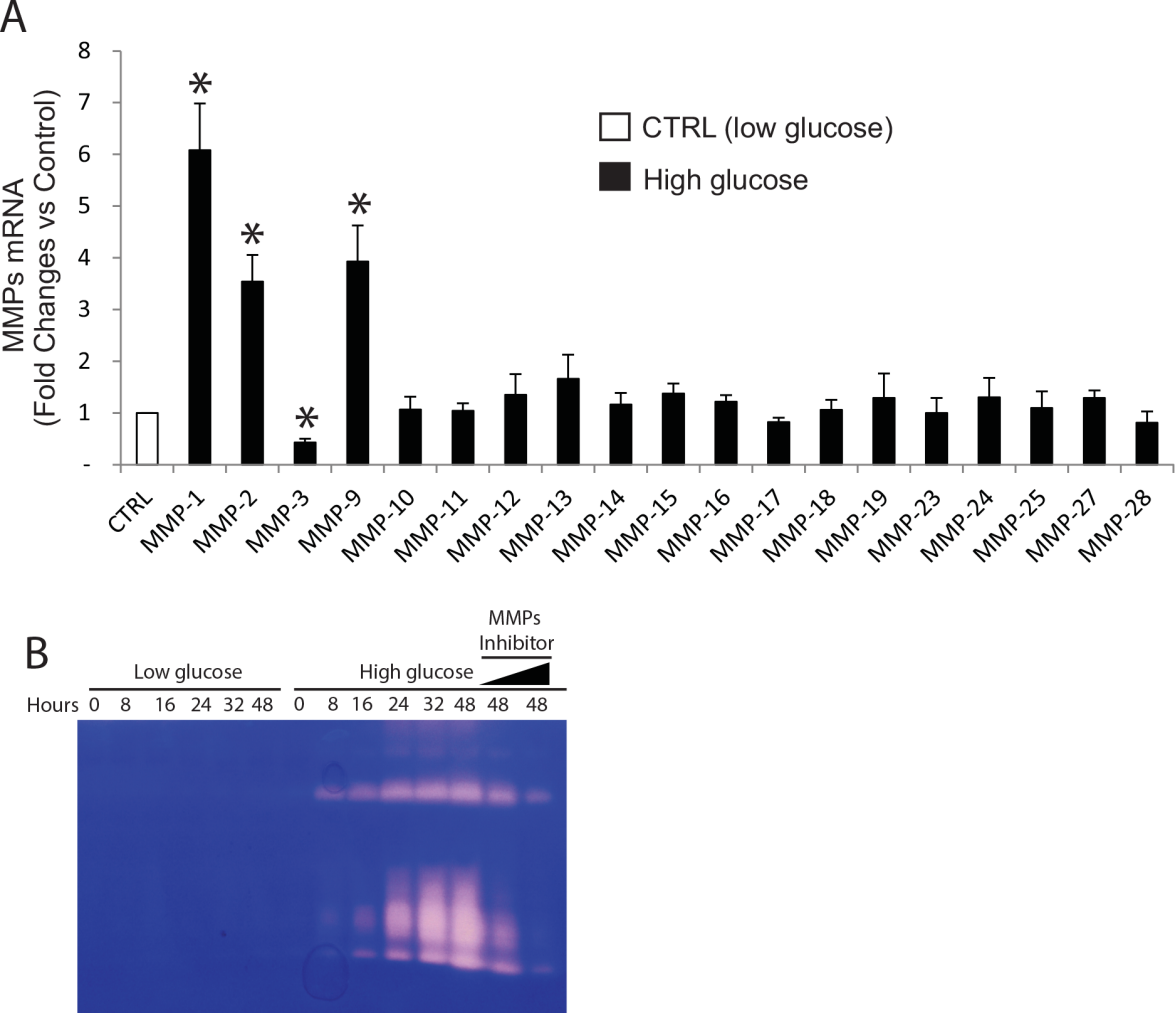
The effect of high glucose culture on MMPs mRNA levels and proteolytic activity in primary human skin dermal fibroblasts. Cell were cultured for 48 h in low (5mM) or high (25 mM) glucose media (see Methods for details). The effects of high glucose on the levels of MMPs mRNA expression were quantified by real-time RT-PCR. MMPs mRNA levels were normalized to the housekeeping gene 36B4, as an internal control for quantification. Primary human skin dermal fibroblasts do not express MMP-8 and MMP-20. Mean±SEM. N = 4. *p<0.05. Proteolytic activities from the conditioned media were examined by zymography, as described in “*Methods*”. Areas of protease activity will appear as clear bands against a dark blue background where the protease has digested the substrate. Data are representative of three experiments. MMPs inhibitor (GM60001) was used as specificity of MMPs-mediated proteolytic activity (last two lanes).

Finally, we explored the potential mechanism of elevated MMPs in diabetic human skin dermis. To achieve this goal, human skin dermal fibroblasts, the major cell type in the dermis and responsible for collagen homeostasis, were isolated from human skin samples, and were incubated in low (5mM) and high glucose (25 mM)-containing medium. From this experiment, we confirmed that MMP-1 and MMP-2, which is elevated in diabetic human skin dermis (Fig 1), were significantly induced by high glucose treatment (Fig 5A). In addition to MMP-1 and MMP-2, MMP-9 (gelatinase-B) was also elevated whereas MMP-3 was reduced upon high glucose exposure. Moreover, using casein zymography assay, we confirmed that increased proteolytic activities of MMPs in the conditioned media after high glucose treatment (Fig 5B). This high glucose-mediated proteolytic activity was blocked by a MMP inhibitor (GM60001, Fig 5B last two lanes). These data suggest the potential mechanism in which elevated MMPs expression could result from high glucose related with diabetic human skin dermis.

## Discussion

MMPs comprise a large family of proteinases that are capable of degrading all ECM proteins [25]. According to their substrate specificity, these MMPs are divided into six subclasses: collagenases, gelatinases, stromelysins, matrilysins, membrane-type MMPs and others [26]. Studies conducted by our team and others over the past several years have shown that MMP-1 (collagenase) is significantly elevated in both naturally-aged skin and in sun-induced premature skin aging (photoaging). [27–29]. MMP-1 is the major protease capable of initiating fragmentation of native fibrillar collagen, predominantly type I and III collagens [10, 30]. MMP-1 cleaves collagen fibrils at a single site within its central triple helix. Once cleaved by MMP-1, collagen can be further degraded by other MMPs [28, 31]. Exposure of sun-protected young human skin to purified human MMP-1 in human skin organ culture causes collagen fragmentation, and alterations in the structure and organization of collagen fibrils in the dermis that resemble those observed in aged skin [32].

In this study, we found that MMP-1 is significantly elevated in diabetic human skin. In addition to MMP-1, MMP-2 (gelatinase A), the major protease that digests denatured collagen, is also significantly elevated in diabetic human skin. Furthermore, LCM indicates that both MMP-1 and MMP-2 are primarily produced by dermis in diabetic human skin. These data support the concept that the combined actions of MMP-1 and MMP-2, which are constitutively elevated in diabetic skin, likely lead to chronic, progressive alterations of dermal collagenous ECM, and thus contribute to old-looking skin in diabetes.

Interestingly, compared to non-diabetic human skin aging, in which expression of TIMP-1 is significantly increased [33], elevated MMPs in diabetic skin are not accompanied by alterations of TIMPs expression. The MMPs are inhibited by specific endogenous TIMPs, which comprise a family of four protease inhibitors [34]. All MMPs are inhibited by TIMPs once they are activated except for gelatinases (MMP-2 and MMP-9). TIMP-2 can form a complex with latent MMP-2, and facilitates the activation of latent MMP-2 [35]. Lack of TIMP induction partially explains the preferential induction of MMPs activity in diabetic skin. It is tempting to speculate that the absence of TIMP induction and constitutively elevated MMPs activity in diabetic dermis could be a major driving force for old-looking skin by progressive damage of dermal ECM.

The above speculation is evidenced by significant alterations of dermal collagen structure, characterized by nanoscale fragmentation and disorganization of collagen fibrils in diabetic skin. These alterations of collagen integrity could result in changes in mechanical properties. We do find that fragmented and disorganized collagen fibrils in diabetic skin are stiffer and harder than intact and well-organized collagen fibrils in non-diabetic skin. Elevated LOX expression and highly cross-linked collagen fibrils in diabetic skin may also contribute to alterations of mechanical properties, which is essential to maintaining the tensile and elastic features of the connective tissues.

A wealth of evidence indicates that a tissue’s mechanical microenvironment functions as a critical determinant for a variety of cellular processes including signal transduction, gene expression, and tissue homeostasis [32, 36–38]. One important finding of our study is that there are alterations of the dermal collagen mechanical properties in diabetes. Although the functional significance of the changes in mechanical properties in diabetic skin is not clear, we previously reported that fragmented collagen is unable to support normal cell morphology and mechanical tension within fibroblasts, and this loss of cell shape and mechanical tension is closely associated with increased activity of transcription factor AP-1 [15, 32]. Given that AP-1 functions as a major driving force for expression of multiple MMPs, it is conceivable that AP-1 activity induced by a fragmented collagen microenvironment may contribute to elevated MMPs in diabetic skin. Additionally, fragmented collagen in diabetic skin may also impair mechanical properties of the dermal collagen network, collagen-integrin mechanical sensing, and subsequent integrin signaling events associated with elevated MMPs. Currently, the underlying mechanism associated with the elevations of MMP-1 and MMP-2 as well as LOX is not clear. It is possible that the high glucose conditions of the diabetic skin could positively regulate MMP-1 and MMP-2 as well as LOX expression. Clearly, additional studies are warranted to uncover the precise molecular mechanism(s) by which the factors upregulate MMPs and Lox.

In current study, we found that several MMPs including MMP-1 and MMp-2, which is elevated in human diabetic dermis, induced after high glucose treatment. Some existing literatures reported that high glucose exposure can induce expression of MMPs in several different cell types [39–41], whereas others contradict it [42, 43]. Obviously, the precise mechanism in which glucose metabolism regulates MMPs appears to be complex. It worth mention that elevated MMP production by high glucose treatment in dermal fibroblasts may have important consequences for elevated MMPs and collagen fragmentation, because dermal fibroblasts are major type of the cells responsible collagen homeostasis. The signaling pathways mediating the high glucose regulation of MMPs expression are not completely understood. MMP gene expression is largely regulated by activator protein-1 (AP-1) and nuclear factor-kappaB (NF-kB) at transcriptional level [44]. High glucose has been shown to cause an increase in AP-1 and NF-kB activation [45, 46]. Therefore, it is conceivable elevated AP-1 and NF-kB may drive upregulation of MMPs upon high glucose exposure. It is of interest that high glucose exposure causes opposing effects on MMP-1 and MMP-3 expression although we are unable to detect down-regulation of MMP-3 in diabetic human skin. Discordant regulation of MMP-1 and MMP-3 by high glucose is probably caused by differences in their proximal promoter regions [44]. MMP-1 and MMP-3 both have AP-1 binding sites in their proximal promoter, however, their AP-1 sequences are differ from each other and from the consensus AP-1 sequence. Different AP-1 sequences can bind alternate forms of the AP-1 complex, which can differently regulate their target gene expression [47].

We have previously reported that human skin connective tissue aging is largely caused by aberrant collagen homeostasis [12, 32, 48]. Two interrelated mechanisms are involved; reduced collagen biosynthesis and increased collagen fibril fragmentation. These mechanisms result in a net collagen deficiency, which manifests as thin, fragile skin. Age-related aberrant collagen homeostasis impairs the structural integrity and mechanical properties of the skin, and contributes to age-related skin diseases. In this study, we found that type I and type III collagen mRNA expressions are not changed in diabetic skin, suggesting a unique collagen homeostasis in diabetes, compared to non-diabetic skin aging.

It is worth mentioning that to minimalize the potential environmental risk factors that may affect our endpoints measurement, all diabetic skin and age-matched normal human skin samples are obtained from sun-protected underarm, since ultraviolet from the sun causes our skin to age prematurely (photoaging) [28, 33]. In addition, all subjects included in the study all had no significant skin disorders, such as foot ulcers, psoriasis, and dermatitis, and no the history of smoking. Our study inclusion/exclusion criteria (see Materials and Methods for details) minimalized the environmental risk factors that affect skin. We believe that elevated MMP-1, MMP-2, and LOX in diabetic skin are likely caused by aberrant glucose metabolism, as supported by our in vitro experiment (Fig 5).

Some skin problems are more common in patients with diabetes, such as diabetic foot ulcers, which are a frequent and disabling complication resulting in significant morbidity [49, 50]. Several published studies have shown altered expression of MMPs and TIMPs in the wounds of diabetic patients [51–55]. Despite extensive studies, the molecular processes underlying impaired wound healing in diabetes are still not fully understood. Our finding that chronic and progressive alterations of the dermal collagenous microenvironment due to constitutive elevation of MMPs and LOX may have a profound effect on non-healing in diabetic foot ulceration. The aberrant collagen microenvironment due to elevated MMPs, LOX, and alteration of dermal mechanical properties could serve as a risk factor for diabetic foot ulceration. Understanding the quality of diabetic skin may help us to predict those who are predisposed to the development of diabetic foot ulceration and other skin problems in diabetes.

## Conclusion

**Fig 6:**
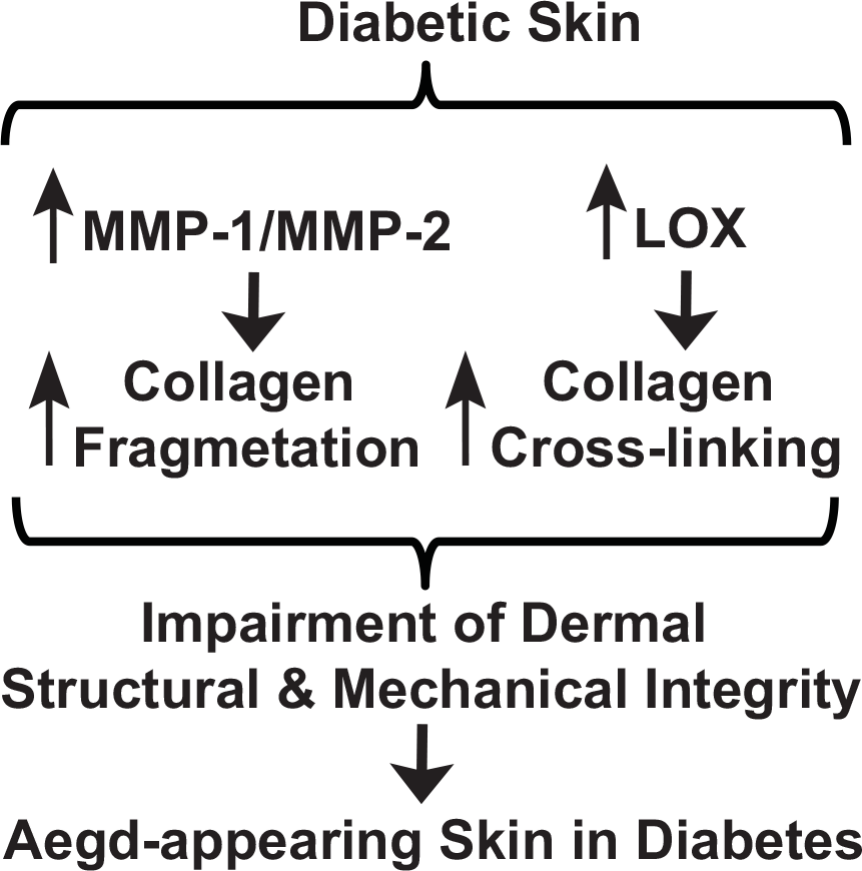
Proposed model for alterations of dermal collagen structural and mechanical properties contribute to old-looking skin in diabetes. Diabetic dermis constitutively expresses elevated levels of MMP-1/MMP-2 and LOX, which result in increased collagen fragmentation, crosslinking, and consequently alterations of dermal structural and mechanical properties of the dermis. Constitutive elevation of MMPs and LOX over decades is thought to result in accumulation of fragmented and cross-linked collagen fragments, and thus contribute to aged-appearing skin in diabetes.

We propose a working model for alterations of the structural and mechanical properties of dermal collagen in contribution to the aged appearance of skin in diabetes (Fig 6). Diabetic dermis shows elevated levels of MMP-1/MMP-2 and LOX, which may contribute to increased collagen fragmentation, crosslinking, and consequently alterations of mechanical properties of the dermis. Elevation of MMPs and LOX over the years is thought to result in accumulation of fragmented and cross-linked collagen fragments, and thus may contribute to aged-appearing skin in diabetes. Our data suggest that impaired the structural integrity and aberrant collagen microenvironment may have a profound effect on the development of skin disorders in diabetic patients.

## Abbreviations

MMPs: matrix metalloproteinase family
ECM: extracellular matrix
COL-1: type I collagen
AFM: atomic force microscopy
TIMP: tissue inhibitor of matrix metalloproteinases
LOX: lysyl oxidase
